# Acute Myocardial Infarction with Simultaneous Gastric Perforation

**DOI:** 10.5811/cpcem.2017.2.33433

**Published:** 2017-05-09

**Authors:** Alon Kaplan, Dan Schwarzfuchs, Vladimir Zeldetz, Jing Liu

**Affiliations:** * Ben Gurion University of the Negev, Soroka University Medical Center, Department of Emergency Medicine, Beer Sheva, Israel; † Kern Medical, Department of Emergency Medicine, Bakersfield, CA

## Abstract

Acute myocardial infarction and perforated peptic ulcer disease with associated peritonitis are both medical emergencies requiring urgent intervention. This patient presented with both emergencies simultaneously. Current literature is devoid of guidance as to which should be addressed initially. A multidisciplinary discussion was conducted leading to a unanimous decision for initiating percutaneous coronary intervention (PCI). After successful PCI, the patient was immediately taken to the operating room for laparoscopic repair of the perforated viscous. Subsequent to the operative repair, the patient became hemodynamically unstable and a repeat electrocardiogram demonstrated complete right coronary occlusion. Shock ensued and the patient died in the intensive care unit despite this plan of care. It is our opinion that this case reveals the need for expert panels to devise decision algorithms for concomitant presentations of life-threatening diseases.

## INTRODUCTION

Acute myocardial infarction with ST-segment elevation is a medical emergency. A preponderance of literature supports that rapid treatment with cardiac catheterization within 90 minutes is associated with lower rates of in-hospital mortality.^1^ This rapid “door-to-balloon” time is also associated with decreased mortality rates at 30 days as well as one year, and has become the standard of care in many healthcare settings.^2^,^3^ Peptic ulcer perforation is a fatal complication of peptic ulcer disease, which occurs in 1.5–7.8 out of 100,000 people per year based on a study conducted among the Swedish population.^4^ The mortality rate associated with peptic ulcer perforation is approximately 10%,^4^ and delayed treatment of peptic ulcer perforation (more than 24 hours between symptom onset and hospital admission) has been established as an independent predictor of 30-day mortality by the peptic ulcer perforation (PULP) score.^5^ When both of these time-sensitive medical emergencies present concomitantly, even experienced physicians may find difficulty in deciding which pathologic process to address first.

## CASE REPORT

A 66-year-old Bedouin woman with a past medical history of uncontrolled type 2 diabetes mellitus, dyslipidemia and hypertension presented to the emergency department (ED) with a chief complaint of abdominal pain for two days. She described the pain as sharp, constant, and located in the epigastric area radiating to her back. It was associated with nausea and vomiting. She denied any history of nonsteroidal anti-inflammatory drug or other analgesic use and had no known history of coronary artery disease or prior surgeries.

**Image 1:**
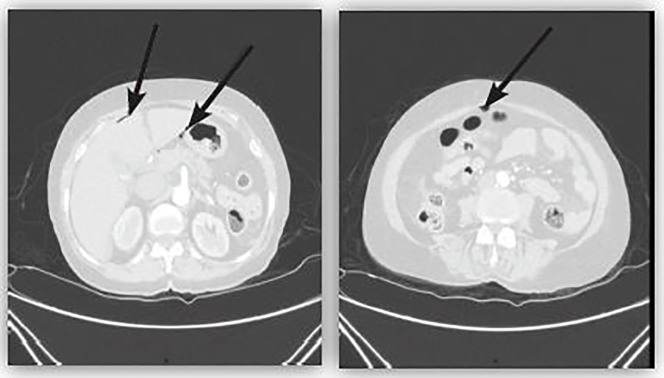
Computed tomography scan showing free air in the abdominal cavity.

**Image 2:**
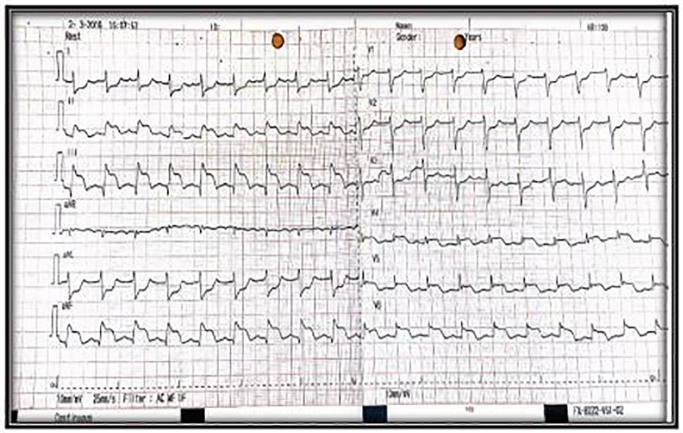
Electrocardiogram shows ST elevations in the inferior and lateral walls.

On presentation the patient appeared stable with normal vital signs. The physical exam was remarkable for a rigid abdomen with diffuse abdominal tenderness. An immediate acute abdominal series was non-diagnostic without evidence of pneumoperitoneum. Analysis of venous blood revealed significant metabolic acidosis (pH 7.29, HCO_3_^−^ 15.6 mmol/L, pCO_2_ 26.2 mmHg, lactate 4 mmol/L). Computed tomography (CT) of the abdomen and pelvis was subsequently performed, which demonstrated free intraperitoneal air suggestive of a perforated viscous (Image 1). After the CT, the patient began to experience new-onset chest pain. An electrocardiogram (ECG) was obtained showing ST-segment elevation in the inferior leads II, III and AvF (Image 2).

Given the concomitant presentation of two emergent pathologies, a multidisciplinary discussion was conducted between the emergency physician, cardiologist and general surgeon to determine the proper course of treatment. They unanimously agreed that cardiac catheterization and percutaneous coronary intervention (PCI) would take precedence over laparoscopic surgical repair of the patient’s perforated viscus. This decision was based on the clinical judgment that an acute STEMI was the more immediate threat to life compared to perforated viscus without evidence of active exsanguination. Consideration was also given to the rapid nature of PCI compared to laparoscopic surgery.

CPC-EM Capsule

What do we already know about this clinical entity?

There is limited literature regarding the treatment of these two diseases occurring simultaneously and there are no guidelines regarding management of such complex patients.

What makes this presentation of disease reportable?

Concomitant acute myocardial infarction and gastric ulcer perforation constitute a challenge both diagnostic and therapeutic leading to increased morbidity and mortality even with prompt diagnosis and treatment.

What is the major learning point?

In unstable patients, the emergency physician should consider another life-threatening diagnosis even in the presence of an ST elevation myocardial infarction.

How might this improve emergency medicine practice?

By maintaining a high index of suspicion for concomitant disease processes, future case reports may provide more clarity on how to approach this complex clinical scenario.

**Image 3:**
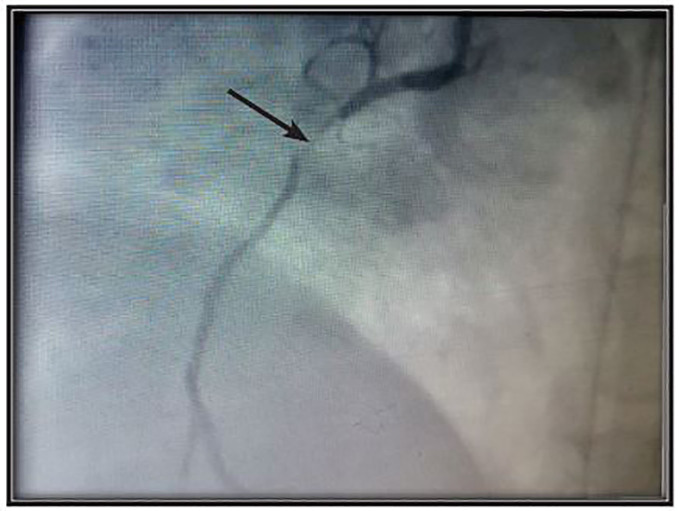
Percutaneous coronary intervention shows an occlusion in the right coronary artery.

The patient received 80mg pantoprazole intravenously (IV) as a temporizing measure for suspected gastric perforation and was rushed to the catheterization laboratory. Prior to PCI she received 3000 units of heparin IV, 300mg of aspirin orally (PO) and 600mg of clopidogrel PO. Catheterization demonstrated significant coronary artery disease. The proximal right coronary artery (with right dominant anatomy) had near total occlusion of the artery (99%), consistent with thrombolysis in myocardial infarction (TIMI) grade 1 flow. Presence of a large coronary thrombus was seen, with minimal anterograde flow beyond the occlusion (Image 3). PCI with a bare metal stent (BMS) insertion was then performed successfully.

The patient was then transferred directly to the operating room (OR) in hemodynamically stable condition. She was sedated and intubated and laparoscopic surgery was initiated. A 2mm perforation was visualized in the prepyloric gastric antrum. The defect was repaired and an omental patch was placed. Operative time was less than 20 minutes. After the operative repair but while still in the OR, the patient became hemodynamically unstable with systolic blood pressure measuring 40mmHg. IV vasopressors were initiated and the patient was transferred to the ICU on a ventilator. She remained hemodynamically unstable 12 hours postoperatively despite resuscitative measures and maximal vasopressor support.

**Image 4:**
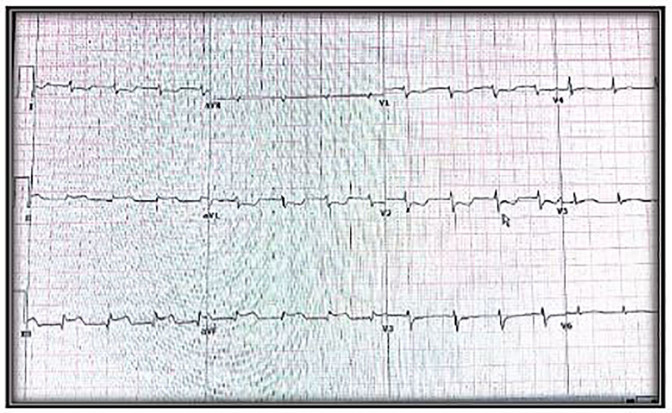
Electrocardiogram shows ST elevation in the inferior wall.

A repeat ECG was obtained, which again showed ST-segment elevation (Image 4). The patient was then taken for a second cardiac catheterization, which revealed a total occlusion of the right coronary artery due to early subacute stent thrombosis. The thrombus was partially evacuated but could not be completely removed. An additional BMS was inserted and the patient was transferred to the intensive coronary care unit where she continued to be hemodynamically unstable. The patient subsequently passed away an hour later.

## DISCUSSION

Acute MI occurring simultaneously with gastric ulcer perforation is an uncommon scenario that can have fatal consequences. Even with prompt diagnosis and treatment, complications arising from these two disease processes are associated with increased morbidity and mortality.^1^–^3^,^5^,^6^ There is limited literature regarding the treatment of these two diseases occurring simultaneously, and available literature consists mainly of case reports. Currently, there are no guidelines regarding management of such complex patients. Differentiating between STEMI and perforated viscus can also be quite challenging. While STEMI can manifest clinically with epigastric complaints, perforated viscus can also be accompanied by ischemic changes on ECG including ST-segment elevation.^7^ A similar case was described in 1967, and the authors suggested that the association may be more common than previously thought. Without a high suspicion for cardiac pathology, these cases may easily be misdiagnosed as the result of peptic ulcer perforation alone.^8^ Another report regarding concomitant perforation and acute MI suggested performing surgical treatment first; however, they do not cite any literature supporting this decision.^9^

A decision analysis in patients with acute MI and upper gastrointestinal (GI) bleeding has been proposed, resulting in esophagogastroduodenoscopy (EGD) prior to PCI as a strategy with better outcomes, but the distinction between non-STEMI and STEMI was not made in this study.^10^ In a case of a patient with upper GI bleeding and acute MI, it is reasonable to perform EGD prior to PCI since the anticoagulation necessary to perform PCI has the potential to worsen GI bleeding. While this may be a reasonable approach, it is important to keep in mind that MI has higher rates of 30-day mortality as well as higher rates of in-hospital mortality.^11^ It is also important to recognize upper GI bleeding and perforated gastric ulcers as distinct entities with differing prognosis and treatment. For example, it has been shown that ulcer perforation has higher mortality rates compared to upper GI bleeding especially in the elderly population.^12^

While there is lack of data regarding whether surgical repair of a perforated gastric ulcer or PCI for STEMI should come first, 1 there is comprehensive data supporting the superior outcomes of early revascularization for STEMI. Based on this, it is our opinion that PCI should be performed prior to surgical repair in patients presenting with simultaneous disease processes. Due to the rarity of this scenario, as well as ethical concerns, there is no option for a randomized clinical trial to compare the two approaches. By maintaining a high index of suspicion for concomitant disease processes, future case reports may provide more clarity on how to approach this scenario.

## CONCLUSION

The presence of two life-threatening diagnoses occurring simultaneously in a patient is rare but not unheard of. Recognizing the concomitant disease processes is crucial, but determining the most effective sequence of treatment is not always readily apparent. The establishment of a clear algorithm may facilitate the treatment of such patients, such as those who suffer from acute myocardial infarction complicated by another medical emergency. We therefore suggest that an expert panel use available literature and expert opinion to devise a decision algorithm for future cases in order to provide optimal treatment for these complex cases.
